# Management of Secondary Metabolite Synthesis and Biomass in Basil (*Ocimum basilicum* L.) Microgreens Using Different Continuous-Spectrum LED Lights

**DOI:** 10.3390/plants13101394

**Published:** 2024-05-17

**Authors:** Mohammad Reza Fayezizadeh, Naser Alemzadeh Ansari, Mohammad Mahmoodi Sourestani, Masayuki Fujita, Mirza Hasanuzzaman

**Affiliations:** 1 Department of Horticultural Science, Faculty of Agriculture, Shahid Chamran University of Ahvaz, Ahvaz 61357-43311, Iran; 2 Faculty of Agriculture, Kagawa University, Kagawa 761-0795, Japan; 3 Department of Agronomy, Faculty of Agriculture, Sher-e-Bangla Agricultural University, Sher-e-Bangla Nagar, Dhaka 1207, Bangladesh; 4 Kyung Hee University, 26 Kyungheedae-ro, Dongdaemun-gu, Seoul 02447, Republic of Korea

**Keywords:** biomass, carotenoids, flavonoids, light quality, plant antioxidants, vitamin C

## Abstract

Different LED light spectra (LS) are absorbed by different plant photoreceptors and can control biomass and plant secondary metabolite synthesis. In this study, the effects of continuous-spectrum LED lights (red, blue, white, red + blue, and 12 h blue + 12 h red) on the production value, antioxidant compounds, and biomass of basil (*Ocimum basilicum* L.) microgreens (Red Rubin, Violeto, and Kapoor cultivars and the Ablagh genotype) were investigated. The results showed significant effects of LS on cultivar (Cv) and the interaction of LS and Cv on the studied traits. The highest quantitys of chlorophyll a, total chlorophyll, and nitrate were obtained in Violeto under blue lighting. Red lighting enhanced starch synthesis in Red Rubin and flavonoids in the Violeto Cv. The highest biomass (4.54 kg m^−2^) was observed in the Ablagh genotype and the highest carbohydrate synthesis in Violeto Cv in the red + blue treatment. The highest anthocyanin content (26.33 mg 100 g^−1^ FW) was observed for Red Rubin Cv under 12 h blue + 12 h red light. The greatest antioxidant capacity (83.57% inhibition), the highest levels of phenolic compounds (2027.25 mg GA 100 g^−1^ FW), vitamin C (405.76 mg 100 g^−1^ FW), proline, antioxidant potential composite index (APCI), and the greatest production values were obtained for the Ablagh genotype under blue lighting. Taken together, the experiment findings indicate that growing the Ablagh genotype under continuous blue lighting can increase the antioxidant capacity, phenolic compounds, and vitamin C and that this LED light spectrum can be used as a practical method to produce basil microgreens with high nutritional health value.

## 1. Introduction

Light is one of the most important environmental factors controlling plant growth. Not only is light the source of energy for carbon fixation in plant photosynthesis, but the light spectrum (LS) also alters plant morphology and the synthesis of secondary metabolites (SEs) through regulatory effects on morphogenesis, physiological metabolism, growth and development, gene expression, total antioxidant capacity (TAC), and enzyme activity [1]. The most important LS used in today’s plant factories are blue (B) and red (R) light, as these spectra have the greatest effect on photosynthesis, the accumulation of TAC, and the production of pigments through their effects on stomatal opening, electron transfer, and Rubisco activity [2]. The use of LEDs as the primary light source in plant production factories allows customization of the LS to meet plant goals and optimize biomass and SE production [3].

Plants absorb over 90% of different LS through photomorphogenic photoreceptors, like phytochromes, phototropins, and cryptochromes [4]. These photoreceptors react differently to light intensity and wavelength to affect the synthesis of plant biochemical and control physiological responses and play crucial roles in the overall functioning of plants [5]. For example, plant cryptochromes influence processes like stomatal opening, biomass production, and the biosynthesis of anthocyanins (ACNs), carotenoids (CARs), and chlorophyll (Chl). Different LS activate enzymes involved in secondary metabolite biosynthesis pathways, such as PAL (phenylalanine ammonia-lyase) in the phenylpropanoid pathways, thereby increasing the accumulation of bioactive compounds in plants and making LS an essential factor controlling many biochemical pathways necessary for plant growth and development [6]. 

Previous studies have shown that different factory-grown microgreen species respond differently to LS with different R/B ratios. Some microgreens exhibit a limiting crop biomass under B light, while others, like onion, red marigold, borage, green basil, and peas experience limited growth under R light [7]. Growth of lettuce under B light can regulate the production of various compounds, including vitamin C, proteins, and total flavonoid compounds (TFCs), and increase CARs [8]. Higher B light levels also positively affect the accumulation of total phenolic compounds (TPCs) and ACNs in Brassicaceae microgreens [9]. B light exposure increases CARs in beet microgreens, while decreasing CARs in parsley and mustard microgreens [5]. In basil plants, the choice of LEDs significantly influences the contents of vitamin C, Chl, soluble sugar, and protein, as well as the TAC activity [10]. Vaštakaitė et al. [11] reported that R light affected TPC and TAC activity, while B light enhanced the growth, vitamin C, total phenols, ACNs, TFCs, and TACs in green basil. Lobiuc et al. [12] showed that growing basil microgreens under B light enhanced their growth, biomass, and Chl and ACN contents, while applying R light to the green Cv and B light to the red Cv increased the TPC and TAC levels.

Basil (*Ocimum basilicum* L.) microgreens are widely cultivated in plant production factories due to their compatibility with floating systems and controlled environments. These microgreens are known for their high genetic diversity and TAC potential. Among 21 studied Cv, the Violeto, Red Rubin, Kapoor, and Ablagh genotypes have shown the highest TAC potential [13]. Different treatments, such as nutrient solution concentrations and short and long photoperiods, can alter the antioxidant properties of factory-grown plants [14]. However, the overall response of plants to different LED LS in terms of desirable metabolites is species-specific, and regulating the production of specific TACs using LEDs is complex due to the presence of redundant metabolic pathways. The aim of the present study was to investigate the effects of different continuous lighting (CL) LED spectra on the biomass, TAC, and biochemical compound production in basil microgreens, with the overall goal of identifying the best balance between biomass and LS. Four different basil microgreen Cv grown in a floating system under CL were also compared for their production values.

## 2. Results

### 2.1. Photosynthetic Pigments

The simple effects and the interaction of LS and Cv on the Chl *a*, total Chl, and CARs showed significant differences at the 1% level. The interaction of LS and Cv on the amount of Chl *b* was significantly different at the 5% level, but the simple effects did not show significant differences. The highest quantity of Chl *a* and total Chl were observed in the Violeto Cv under B light. The highest quantity of Chl *b* was observed in the Ablagh genotype under the B:R (12/12 h) treatment. The highest quantity of CARs was measured in the Violeto Cv under R light (Table 1). The total Chl level showed a positive and significant correlation with the APCI, production value, and nitrate content, while the total Chl content was negatively correlated with the starch level. The CAR levels showed positive and significant correlations with TFC, APCI, and starch levels (Table 2).

### 2.2. Fresh Biomass

The simple effects and the interaction of LS and Cv on biomass were significantly different at the 1% level. The highest fresh biomass was measured in the Ablagh genotype grown under RB light, which showed an increase of 171.85% compared to the Red Rubin Cv grown in W light (Table 1). The biomass of basil microgreens was positively and significantly correlated with TACs, production value, and nitrate content (Table 2). 

### 2.3. Vitamin C

The simple effects and the interaction of LS and Cv on the amount of vitamin C showed significant differences at the 1% level. The highest quantity of vitamin C was measured in the Ablagh genotype grown under B light, which showed an increase of 164.70% compared to the Red Rubin Cv grown under RB light (Table 3). Vitamin C was positively and significantly correlated with APCI, production value, proline, and carbohydrates (Table 2).

### 2.4. Total Antioxidant Capacity (TAC)

The simple effects and the interaction of LS and Cv on TAC were significantly different at the 5% level. The highest TAC was measured in the Ablagh genotype grown under B light, which showed an increase of 144.50% compared to the Red Rubin Cv grown in R light (Table 3). TACs were positively and significantly correlated with TPCs, TFCs, vitamin C, production value, nitrate, and proline (Table 2).

### 2.5. Total Phenolic Compounds (TPC)

The simple effects and the interaction of LS and Cv on the total TPCs were significantly different at the 1% level. The highest quantity of total TPCs was found in the Ablagh genotype grown under B light, which showed an increase of 175.10% compared to plants grown under R light (Table 3). TPCs were positively and significantly correlated with TFCs, vitamin C, APCI, production value, and proline (Table 2). 

### 2.6. Total Flavonoid Contents (TFC)

The simple effects and the interaction of LS and Cv on the amount of TFCs were significantly different at the 1% level. The highest quantity of TFCs was observed in the Violeto Cv grown under B light, which showed an increase of 616.82% compared to plants grown under R light (Table 3). TFCs were positively and significantly correlated with vitamin C, APCI, production value, proline, carbohydrate, and starch (Table 2).

### 2.7. Anthocyanins (ACNs)

The simple effects and the interaction of LS and Cv on the amount of ACNs were significantly different at the 1% level. The highest quantity of ACNs was observed in the Red Rubin Cv grown under B:R (12/12 h) light, which showed an increase of 385.80% compared to the Kapoor Cv grown under R light (Table 3). ACNs were positively and significantly correlated with the nitrate level (Table 2).

### 2.8. Nitrate

The simple effects and the interaction of LS and Cv on the nitrate content were significantly different at the 1% level. The highest amount of nitrate was observed in the Violeto Cv grown under B light, which showed an increase of 280.26% compared to the Ablagh genotype grown under R light (Table 3). The nitrate level was negatively and significantly correlated with the starch level (Table 2).

### 2.9. Proline Content

The simple effects and the interaction of LS and Cv on proline were significantly different at the 1% level. The highest amount of proline was measured in the Ablagh genotype grown under B light, which showed an increase of 358.28% compared to the Red Rubin Cv grown under RB light (Table 3). Proline had a positive and significant correlation with carbohydrates (Table 2).

### 2.10. Soluble Carbohydrate Content

The simple effects and the interaction of LS and Cv on the amount of carbohydrates were significantly different at the 1% level. The highest quantity of carbohydrates was determined in the Violeto Cv grown under RB light, which showed an increase of 209.50% compared to the Cv Red Rubin grown under the same LS treatment (Table 3). Carbohydrate levels were negatively and significantly correlated with starch levels (Table 2).

### 2.11. Starch Content

The simple effects and the interaction of LS and Cv on the amount of starch were significantly different at the 1% level. The highest amount of starch was obtained in the Red Rubin Cv grown under R light, which showed an increase of 65.43% compared to the Violeto Cv grown under B light (Table 3). 

### 2.12. Antioxidant Potential Composite Index (APCI)

**Figure 1:**
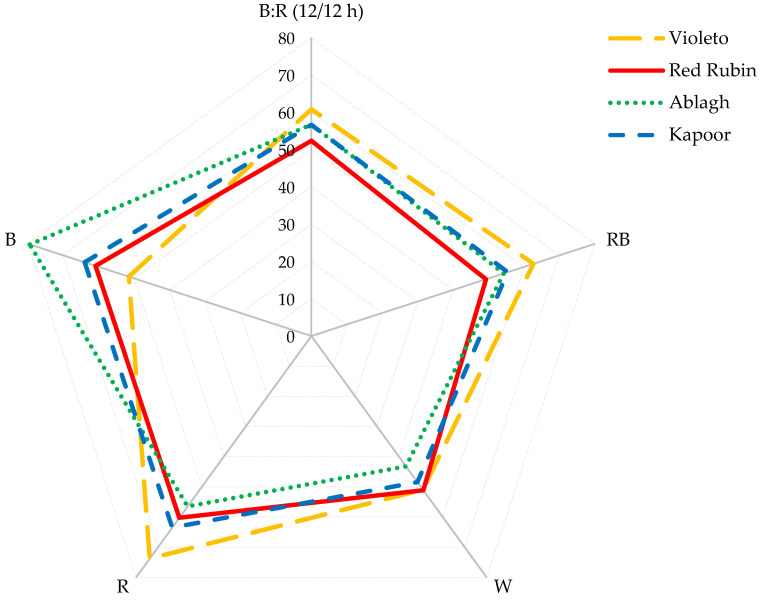
The APCI of the different basil microgreen cultivars under different LED light spectra. B: R (12/12 h) = Blue light 12 h + Red light 12 h, RB = Red + Blue continuous light, W = White continuous light, R = Red continuous light, B = Blue continuous light.

The simple effects and the interaction of LS and Cv on APCI were significantly different at the 1% level. The highest APCI value was measured in the Ablagh genotype grown under B light, which showed an increase of 84.81% compared to plants grown under W light (Figure 1).

### 2.13. Production Value

**Figure 2:**
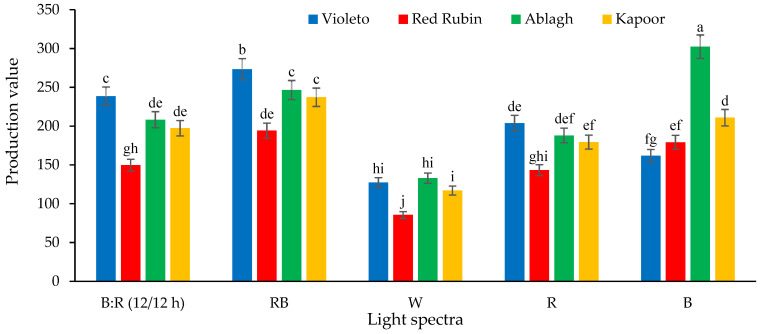
The production value of different basil microgreen cultivars under different LED light spectra. The letters marked in the figure indicate the significance of the difference (*p* ≤ 0.05, Duncan’s test). B: R (12/12 h) = Blue light 12 h + Red light 12 h, RB = Red + Blue continuous light, W = White continuous light, R = Red continuous light, B = Blue continuous light.

The simple effects and the interaction of LS and Cv on production value were significantly different at the 1% level. The highest production value was observed in the Ablagh genotype grown under B light, which showed an increase of 253.63% compared to the Red Rubin Cv grown under W light (Figure 2).

### 2.14. The Balance between APCI and Biomass

**Figure 3:**
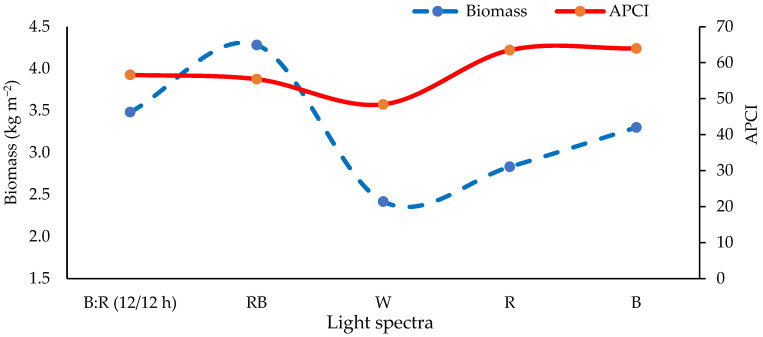
Changes in biomass and APCI of four basil microgreens under different LED light spec-tra. B: R (12/12 h) = Blue light 12 h + Red light 12 h, RB = Red + Blue continuous light, W = White continuous light, R = Red continuous light, B = Blue continuous light, APCI = Antioxidant potential composite index.

As shown in Figure 3, the best balance between biomass and APCI was obtained with the RB light treatment, whereas the poorest balance rate was obtained with W light.

## 3. Discussion

Chls and CARs (comprising the two classes of carotenes and xanthophylls) are plant SEs that function as antenna pigments to protect photosynthetic components from harm due to light exposure [6]. The CAR/Chl ratio increases under excessive light conditions and can limit the damage caused by excess light, including CL, and represents one of the defense mechanisms activated in basil microgreens that lead to resistance and continued growth without damage under CL conditions. Energy dissipation by non-photochemical quenching (NPQ) during the conversion of violaxanthin, antheraxanthin, and lutein epoxide to zeaxanthin and lutein by light-harvesting antenna proteins is another way that xanthophylls can offer photoprotection [15]. Light-responsive genes can control the production of Chl and CAR pigments, even if the accumulation of CARs and other pigments is genetically controlled in many plant parts and fruits [16,17]. For example, the growth of grapes under B light has been shown to upregulate the expression of eight important gene products involved in chloroplast biosynthesis [16]. 

In microgreens like basil, the influence of light quality on the accumulation of CARs remains poorly understood, as light effects vary according to the species and the combination and kind of light [18]. Very little information is available, although CARs and xanthophylls, including lutein, antheraxanthin, violaxanthin, and beta-cryptoxanthin, are known to increase with the use of LEDs [19]. According to Llorente et al. [20], the R LS directly regulates the activation of the phytoene synthase (PSY) gene, which encodes the rate-limiting enzyme in the CAR biosynthesis pathway. Furthermore, the R LS also has the ability to control the expression of CAR genes [21]. The results of the present study demonstrate that the genetic influence may become apparent in the way that photosynthetic pigments respond to different LS. Specifically, the total Chl of the Red Rubin Cv increased significantly under B light compared to R light (where it showed the lowest value). Thus, the CL stress imposed during basil microgreen cultivation can be attenuated by growth under B LS to increase the amount of total Chl, while the R LS can be used to increase the amounts of CARs. The result will be an increase in the energy trapped by photosynthesis and a boost in the TAC levels, as well as a consequent improvement in microgreen biomass.

Fresh biomass is regarded as one of the most crucial factors in the microgreen growing business because biomass production is a major limitation in microgreen plant factories. The diversity of microgreen species and the applied LS determine how light affects fresh weight. Since Chl pigments absorb R and B LS more efficiently than other parts of the spectrum, both types of light are useful for promoting plant development [22]. Consequently, adding a combination of R and B LED light would be expected to improve basil microgreen biomass. In contrast to the findings of Alrajhi et al. [23], who found no effect of combined RB LS in lettuce, the combination of RB LS in the present study enhanced the development and fresh biomass of basil microgreens. The variation of LS effects across plant species is likely due to genetic differences in pigment compositions, photoreceptor types, plant morphology, and biochemical compounds, which are believed to be the genetic factors influencing these species responses. Our experiment showed that RB LS under CL conditions can enhance the biomass of basil microgreens, making this a practical method for producing these plants.

Increases in TAC molecules, including phenols, which function as both light-protectors and TAC in the photosystem, may be linked to changes in the LS [24]. The B LS in the present raised the TAC and the concentration of total TPC when compared to the other LS treatments. Numerous plant species have demonstrated that light quality can influence the generation of ROS and TAC [25]. According to El-Esawi et al. [26], expression of transcription factor (HY5) can accelerate the effects of B and R LS on ROS through cryptochromes and phytochromes. Consequently, changes in ROS concentration that are reliant on light quality can also alter TAC levels. In the present study, utilization of the B LS generally increased the capacity of TACs, including non-enzymatic TACs like vitamin C and TPCs. Therefore, using the B LS to produce TACs in basil microgreens in a floating system can be a successful strategy. 

Plants produce a significant class of SEs known as TPCs, which are primarily divided into phenolic acids and polyphenols. The shikimate pathway produces phenylalanine, a common intermediate that serves as the key substrate for PAL, an important enzyme that converts phenylalanine into TPCs [27]. Research on the impact of LED LS on key enzymes involved in basil microgreen polyphenol production is limited, despite several investigations on TPC measurement. The use of B LS treatment by increasing the synthesis of L-phenylalanine can increase the synthesis of simple phenols such as cinnamate and coumarate [28]. This process is enhanced in response to B LS treatment, facilitating the manufacture of phenolic compounds. Furthermore, the synthesis of TPC is aided by an increased production of phenylalanine over tryptophan, suggesting that LEDs may regulate TPCs both directly by promoting the expression of important enzymes and indirectly by increasing precursor molecule production [29,30]. Lobiuc et al. [12] reported that the use of RB LS in growing red basil microgreens can lead to a 4 to 5 fold increase in the synthesis of caffeic acid and rosmarinic acids respectively, compared to W light. Taulavuori et al. [31] found that B LS increased phenolic biosynthesis through the phenylpropanoid pathway, in agreement with the present findings that B LED light played a key role in increasing the synthesis of TPCs. Taken together, our results indicate that growth of the Ablagh genotype under continuous B LS would boost TAC levels and raise TPC levels, which are the most abundant plant TACs.

TFCs are widely distributed SEs in plants and are involved in several processes, such as defense against pathogens, color, stress responses, and the protection of leaf cells against oxidative damage [32]. Both the accumulation of TFCs and the expression of genes involved in their manufacture in plant species are influenced by light quality [33], and several previous studies have reported a key role of LED use in the biosynthesis of TFC in plants [34]. The increased expression of genes encoding enzymes involved in the phytochemical biosynthesis of TFCs, such as PAL, chalcone synthase (PHS), CHI, and flavonol synthase, in basil microgreen leaves was reported as the reason why the plants grown under B LS synthesized more TFCs than those grown under other conditions [35,36]. In the present study, the Violeto Cv had the highest TFC content under B light and the lowest TFC content under R light, confirming the general variations in Cv responses to LS differences. Overall, our findings indicate that the B LS should be used to a greater extent than other colors to increase TFCs and improve the antioxidant properties of basil microgreen plants.

Another important antioxidant is vitamin C (ascorbic acid), which is involved in many processes related to growth and development, including the production of plant hormones, iron absorption, photosynthesis, cell division, elongation, and differentiation, as well as defense against environmental stressors. The L-galactose route is the primary mechanism for ascorbate synthesis in plants [37]. While darkness causes vitamin C catabolism, increasing the photoperiod can regulate vitamin C storage through several mechanisms [38]. The use of B LS enhanced the synthesis of vitamin C in basil microgreens, in agreement with a previous study by Chen et al. [39], who found that both B LS and R LS alone boosted the ascorbic acid concentration of lettuce. Massot et al. [40] reported that the light quality of LEDs affected the activity of enzymes involved in the production and oxidation of vitamin C, thereby regulating the concentration of vitamin C. Specifically, B LS treatment induced the transcriptional upregulation of L-galactose pathway genes involved in vitamin C biosynthesis [41]. In the present study, the quantity of carbohydrates increased under conditions in which vitamin C was maximal (e.g., in the Ablagh genotype grown under B LS or in the Violeto Cv grown under RB LS). Due to their close relationship, photosynthesis and vitamin C production are influenced by light. According to Loi et al. [17], soluble carbohydrates serve as both the primary end product of photosynthesis and a precursor of vitamin C. Basil microgreen nutritional and antioxidant qualities can therefore be enhanced by growth under a B LS, by impacting the production of vitamin C.

ACNs, as a subclass of TFCs, are pigments that give plants their red, purple, and blue hues. They are typically linked to sugars as glycosides and are essential for photosynthesis and for protection against stress due to CL [42]. ACN accumulation requires light, and several spectral characteristics are known to affect ACN synthesis. In particular, research has highlighted the significance of B LS in the modulation of ACN synthesis [43]. The synthesis of ACN pigments varied in the basil microgreen Cv used in the present study, indicating that this pigment production is genetically based on and unique to green and red plant varieties. Cryptochrome (Cry1) perception of the B LS stimulates genes that synthesize ACNs [44]. The synthesis of ACN can increase under B light conditions, and R light can decrease the synthesis of ACN [45]. A previous study revealed that R LS caused a 40% reduction in ACNs in red lettuce [46]. In the present study, the Red Rubin Cv grown in the B:R (12/12 h) condition produced the highest quantities of ACNs. This indicates that the B LS is more effective than the R LS in ACN biosynthesis and that the R LS destroys them. Because ACNs have antioxidant qualities, treating the Red Rubin basil microgreen Cv with a B:R (12/12 h) light regime could boost its ACN production and enhance its aesthetic appeal. 

The synthesis of secondary metabolites, particularly nitrogen molecules like nitrate, is impacted by light spectra [6]. Previous studies have also demonstrated that the application of B LS to leafy greens raises the overall plant nitrogen content and increases the amount of nitrate stored in the leaves. The primary source of nitrogen for plant growth and development is nitrate, which builds up in cell vacuoles to function as an osmotic regulator when nitrogen levels are sufficiently high [47]. Light controls the expression of the nitrate reductase (NR) enzyme, which in turn controls the regulation of nitrate metabolism. The present data unequivocally demonstrate that growth under R LS reduced nitrate content. This process involves the activation of phytochromes PhyA and PhyB and could explain the lower nitrate levels under R light [48,49]. Growth under R LS has also been shown to increase carbohydrate synthesis and the supply of ferredoxin and NADPH, which are used to reduce nitrate in leaves [50]. This is one way that NR regulates nitrate reduction in plants at the post-translational level [51]. The nitrate content in the present study was lower than the maximum levels for nitrates in leafy vegetables are defined by European Commission Regulations [52]. Growth under the B LS has a positive effect on nitrate content, and since its concentration is below the maximum levels for leafy vegetables, this light treatment can be used to increase the nitrate and proline as a nitrogen reserve in basil microgreens.

One nitrogen-containing metabolite in plants is the amino acid proline, which is produced from glutamate and ornithine by a mechanism that is directly connected to cellular energy via the respiratory electron transport chain [53]. The findings of the present study demonstrated that proline production rises in the response to B LS. The Ablaq genotype showed the highest quantity of proline under B light, which indicates that this genotype is stressed under these conditions. In addition to proline, the Ablaq genotype under B light was able to show a high level of TPC, TAC, vitamin C and ACN content, which by synthesizing these compounds along with proline was able to show resistance to light stress. The use of B LS in growing basil microgreens under constant light can be a method to increase TACs and light stress resistance because it increases the biosynthesis of proline in the plant, especially in the Ablagh genotype. This mechanism can be effective as a defense system against the stresses caused by constant light, while also increasing the antioxidant properties and health of the plant.

The ability of the LS to regulate the activity of Calvin–Benson cycle enzymes also makes light a significant regulator of the metabolism of carbohydrates [36]. Remarkably, light causes some carbohydrate catabolism enzymes to be suppressed and others to be unregulated. According to Li et al. [54] and Izzo et al. [55], the application of RB LS elevated the activity of the key Calvin–Benson cycle enzyme in pepper and tomato. The plants cultivated with the RB LS showed boosted Rubisco activity, demonstrating the precise impact of various spectral bands and their ratios on Rubisco. In addition, significant increases in GAPDH activity have been reported in tomato plants following monochromatic R LS treatment and significant decreases in response to monochromatic B LS compared to white light [56]. Previous contradictory results demonstrated that plant response to LS is strongly impacted by species and that plant responses in the glucose synthesis cycle depends greatly on the kind of plant, development stage, and environmental factors. The results of prior research [57], as well as the outcomes of the present study, verify the ability of RB light to increase the activity of enzymes involved in carbohydrate biosynthesis, thereby increasing carbohydrate synthesis in basil microgreens. The RB light treatment increased the biomass of basil microgreens by increasing the production of photosynthesis products. The B LS effects demonstrate that stomatal opening under B LS would lower starch content [58]. B LS treatment can therefore help basil microgreen growth by increasing the accumulation of carbohydrates as a precursor to the synthesis of other plant biochemical with antioxidant properties and raising biomass in basil microgreens. This is demonstrated by the decrease in starch content in the B LS treatment, which indicates that the light treatment has promoted the conversion of starch into soluble sugars.

The synthesis of TPCs, TACs, vitamin C, and proline in the Ablagh genotype suggests that it has a higher potential for TAC synthesis than the other Cv and genotypes [13]. In the present research, this genotype was clearly differentiated from the other Cv. The origin of the Ablagh genotype (Tabriz city, N: 48°25′ and E: 38°2′) suggests that the climate has an effect on plant genetics that is favorable for the synthesis of TACs, and additional treatments, such as modulation of nutrient solution concentration, can enhance this potential even further [14]. The findings presented here for basil microgreens demonstrated that when light is combined with other environmental treatments in the cultivation system, the B LS treatment can effectively boost the antioxidant properties of these Cv and can be useful in the production of basil microgreens. Overall, the production value indicates that the Ablagh genotype under B LS is the most favorable option for growing microgreens in floating systems. Consequently, the use of this light treatment can be used as a defense mechanism against CL damage by increasing the synthesis of proline, vitamin C, and TPC, which will increase the biomass, nutritional quality, and resistance to photo-oxidative stresses in basil microgreens.

## 4. Materials and Methods

### 4.1. Plant Materials and Cultivation System

**Figure 4:**
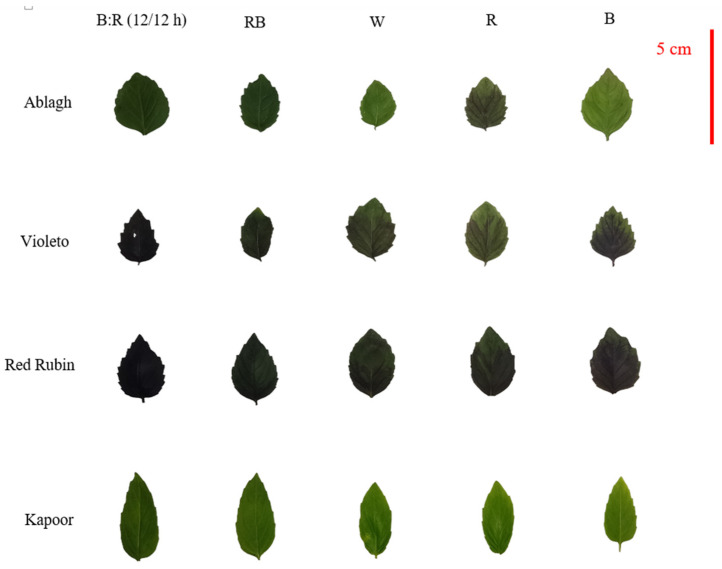
The image of the second leaf of different cultivars of basil microgreen under different light spectra. B: R (12/12 h day/night) = Blue light 12 h + Red light 12 h, RB = Red + Blue continuous light, W = White continuous light, R = Red continuous light, B = Blue continuous light.

In this study, the effects of continuous-spectrum LED lights (red [R], blue [B], white [W], red + blue [RB], and 12 h blue + 12 h red [B:R 12/12 h]) on production value, antioxidant compound contents, and biomass were compared in three cultivars (Violeto, Kapoor, and Red Rubin) and one genotype (Ablagh genotype) of basil microgreens (Figure 4). The study design was a split-plot approach based on a randomized complete block design in a floating system with 4 replications. The seeds were sown in growth trays (105 cells) with 48.5 g m^−2^ density in a mixture of cocopeat and perlite (*v*/*v*: 50%). For faster germination, the trays were incubated at a temperature of 25 °C and 60% humidity for 48 h in the dark. After seed germination, the seedlings were transferred to Hoagland’s nutrient solution (Table S1) with an electrical conductivity of 2.4 mS cm^−1^ and pH 6 ± 0.2 in a floating system [14], under the chosen continuous spectrum LED lighting regimes with a light intensity of 300 ± 15 μmol photons m^−2^ s^−1^. The day and night temperatures of the plant growth chamber were set at 25/21 ± 1 °C, and the humidity during the growth period was 65–70 ± 5%. In order to measure the studied traits, basil microgreens were harvested 25 days after sowing.

### 4.2. Determination of Photosynthetic Pigments

The Chl *a*, Chl *b*, total Chl, and CARs of leaves were measured using a modified Arnon [59] method. Initially, 30 mg of leaves were extracted accurately to four decimal places using 300 μL of acetone 80% and after centrifugation at 3000 rpm for 30 min incubated at 4 °C for 72 h. A microplate reader (INNO, LTEK, Gyeonggi-do, Korea) was used to measure 250 μL of the extract at wavelengths of 663, 645, and 470 nm, and Formulae (1)–(4) were used to calculate photosynthesis pigment contents (mg g^−1^ FW).

Chlorophyll *a* = (((12.7 × A663) − (2.69 × A645))/W) × V (1)

Chlorophyll *b* = (((22.9 × A645) − (4.68 × A663))/W) × V (2)

Total chlorophyll = (((20.08 × A645) + (8.02 × A663))/W) × V(3)

Carotenoids = (1000(A470) − 1.8 (chl *a*) − 58.2 (chl *b*))/198 (4)

W = Sample weight (g), V = Sample volume (mL).

### 4.3. Fresh Biomass

After 25 days of seed sowing, basil microgreens were harvested at the two-leaf stage, and the fresh biomass was reported based on kg m^−2^.

### 4.4. Vitamin C

After 300 mg of leaves was isolated accurately to four decimal places and mixed with 1000 μL of 1% metaphosphoric acid, the vitamin C content was determined by centrifuging the mixture at 850× *g* for 15 min. Subsequently, the supernatant was mixed with 30 ppm of 2,6-dichloroindophenol (DCIP) sodium salt and allowed to incubate for 5 min at 25 °C [60]. In the final step, absorbance at a wavelength of 515 nm was detected using the microplate reader previously mentioned. Using a standard curve for vitamin C (mg Vit C 100 g^−1^ FW); (y = 629.42x × 5.3205, R^2^ = 0.99), the concentration of vitamin C was determined.

### 4.5. Antioxidant Capacity, Polyphenols, Flavonoids, and Anthocyanin

Five hundred mg of fresh leaves was extracted accurately to four decimal places using 500 μL methanol (80%) and stored in the dark for 24 h. For 15 min, the basil extract was centrifuged at 3000 rpm to determine the biochemical and antioxidant substances.

The method used by Sharma and Bhat [61] was used to calculate the TAC. The 126.8 μM DPPH (2-diphenyl-1-picrylhydrazyl) solution was prepared in methanol. Subsequently, 290 μL of the DPPH solution was transferred to a test tube and mixed with 20 μL of the basil leaf extract. The absorbance was scanned at 515 nm while reacting for 16 min. The following equation was used to calculate the percentage of inhibition of DPPH:

Radical scavenging activity DPPH% = {(Abs of control − Abs of sample)/(Abs of control)} × 100 represents the radical scavenging activity.

In order to calculate TPC, 20 μL of the leaf extract was combined with 160 μL of a 1 M Na_2_CO_3_ solution and 20 μL of a 10% (*w*/*v*) Folin–Ciocalteu reagent [62]. With a microplate reader, the absorbance at 765 nm was determined following a 20 min dark incubation period. With the gallic acid calibration curve (y = 105.88x, R^2^ = 0.99), the TPC was calculated in terms of mg gallic acid g^−1^ FW.

To assess TFC, 20 μL of the extracted sample was mixed with 85 μL of distilled water and 5 μL of 5% NaNO_2_. Following a 6 min reaction, the mixture was supplemented with 10 μL of 10% AlCl_3_.6H_2_O. Following an additional 5 min reaction, 20 μL of distilled water and 35 μL of 1 M NaOH were added. The absorbance at 520 nm was recorded and the results were expressed as mg of (+)-catechin (CAE) hydrate g^−1^ FW (y = 875.44x − 55.709, R^2^ = 0.99) [63].

To quantify ACNs, two buffers were created. The first solution was potassium chloride, which had been pH-corrected with HCl to obtain a value of 1 (0.025 M). Sodium acetate, with a pH of 4.5 (0.4 M), was the second solution. Next, after 20 to 50 min, 40 μL of leaf extract and 160 μL of buffer solution were added to the microplate well, and absorbance was measured using a microplate reader at 520 and 700 nm wavelengths [64].

### 4.6. Nitrate Concentration

The nitrate content was ascertained using the modified microplate spectrophotometer method described by Hachiya and Okamoto [65]. For 20 min, a tube holding 50 mg of frozen microgreen powder and 500 μL of deionized water was submerged in boiling water. After that, the sample was centrifuged for 10 min at 3000 rpm. In the next step, a solution containing 10 μL of the extract and 40 μL of salicylic acid in 0.05% (*w*/*v*) sulfuric acid was added to the sample extract. After that, 1 mL of an 8% NaOH (*w*/*v*) solution was added to each sample. The amount of nitrate was calculated using the nitrate standard curve in mg kg^−1^ FW (y = 5149x − 60.696, R^2^ = 0.99), which was detected using a microplate reader at 410 nm. 

### 4.7. Proline Content

A sample of 0.1 g of leaves from each treatment was added to a test tube containing 10 mL of 80% methanol. The test tubes were submerged in 40 °C water for 3 h. To make the ninhydrin reagent, 2.25 g of ninhydrin was dissolved in 54 mL of glacial acetic acid, 36 mL of 6 M phosphoric acid and 450 mL of distilled water. After 50 μL of extract and 250 μL of ninehydrin reagent were added to the microtube, it was incubated for 45 min at 65 °C. After being taken out of the incubator, 250 μL of the solution was added to the microplate reader and absorption was done at 515 nm. The proline content was determined using the L-proline standard curve (y = 4.9804x − 0.0309, R^2^ = 0.99), and given in mg g^−1^ FW [66].

### 4.8. Soluble Carbohydrates Content

Ten ml of 80% ethanol was added to 0.1 g of freshly powdered leaf, and the mixture was centrifuged for 10 min at 5000 rpm. After being transferred the supernatant into Falcon, the prior precipitate was mixed with 10 mL of 80% ethanol and centrifuged for 10 min at 5000 rpm. The 25 μL of the 5% phenol solution was applied to 25 μL solution in the microplate cell, and the microplate cell received 125 μL of pure sulfuric acid. The samples were incubated for 30 min at 25–30 °C. The absorbance of the samples was then determined using a microplate reader at 490 nm. A range of glucose concentrations (g 100 g^−1^ FW) was employed to create the standard curve (y = 4.1363x − 0.0746, R^2^ = 0.99) [67].

### 4.9. Starch Content

The leftover precipitate of the sugar solution was mixed with 260 μL of 52% perchloric acid and 200 μL of cold distilled water. After 15 min of shaking, 400 μL of distilled water was added to the mixture, and it was centrifuged for 10 min at 5000 rpm. After the supernatant was separated out, the remaining residue was mixed with 100 μL of cold distilled water and 130 μL of 52% hydrochloric acid, and the mixture was centrifuged for 10 min at 5000 rpm. The prior mixture was supplemented with the supernatant. For 30 min, the mixture was submerged in an ice bath. After that, 2 mL of distilled water was added to the mixture; 50 μL of the combination solution and 400 μL of 2% anthrone were added to the microtube. After being at 65 °C for 20 min, the samples were moved to an ice bath. Using a microplate reader, the samples were measured at 630 nm [67]. The starch content was determined using the standard curve (y = 78.7930x − 56.0351, R^2^ = 0.99).

### 4.10. Antioxidant Potential Composite Index Calculation

The antioxidant potential composite index was used to measure the antioxidant capacity of basil microgreens [14]. First, individual APCI was calculated by assigning a score of 100 to the highest value in each assay and subsequently calculating the score for all other samples as a percentage of the highest score. Formula (5) was used to calculate APCI, which was the total of six antioxidant activity indices, including TAC, Vit C, Cars, TFC, TPC, and ACNs.

APCI = ((((Measured X1)/(Max X1)) +⋯+ ((Measured X6)/(Max X6)))/N) × 100(5)

X1: Antioxidant capacity; X2: Polyphenols; X3: Vitamin C; X4: Flavonoids; X5: Anthocyanin; X6: Carotenoids; Max: The maximum amount of each antioxidant activity indices; N: Number of the trains (six trains).

### 4.11. Production Value

The production value of basil microgreens was calculated using the biomass multiplying by APCI, which included biomass and all of their antioxidant properties.

### 4.12. Statistical Analysis

IBM SPSS software version 26 was used to analyze the data. The studied trains were analyzed using multivariate analysis of variance (MANOVA) to investigate the combined effect of LED lighting spectra as the main plots and cultivars/genotype as the sub-plots. To compare means, the Duncan multiple range test (*p* ≤ 0.05) was utilized. A bivariate Pearson correlation analysis was performed to see whether there were any linear links between the trains that were being examined.

## 5. Conclusions

Selecting the ideal LS for basil microgreens grown in permanent light can be difficult because of the variety of plant species and Cv. Furthermore, no suitable LS standards have yet been introduced to boost the production of TAC and biomass. By modifying light quality, LED lights can control biomass production and the accumulation of SE in microgreens grown in confined spaces. This study revealed that different varieties of basil microgreens had different responses to light quality, suggesting that commercial varieties differ in their photoreceptor composition. The Ablagh genotype may be favored over other Cv because of its greater potential for synthesis of SE and overall biomass. The use of combined RB LS to increase the biomass and synthesis of TACs can be exploited by producers as a practical strategy for the cultivation of basil microgreens under permanent light. Also, B LS, by increasing the synthesis of TACs such as TPCs, vitamin C and antioxidant capacity, can improve the health properties of basil microgreens.

## Figures and Tables

**Table 1:** Mean comparison of biochemical and antioxidant traits of basil microgreen cultivars and genotypes.

Treatments		Chl *a* (mg g^−1^ FW)	Chl *b* (mg g^−1^ FW)	Total Chl (mg g^−1^ FW)	Carotenoids (mg g^−1^ FW)	Biomass (kg m^−2^)
Light Spectra	Cultivars					
B: R (12/12 h)	Violeto	0.53 ^cdef^	0.18 ^bc^	0.71 ^def^	0.27 ^de^	3.92 ^b^
	Red Rubin	0.53 ^cdef^	0.20 ^bc^	0.74 ^cdef^	0.30 ^cde^	2.86 ^g^
	Ablagh	0.61 ^bcd^	0.31 ^a^	0.93 ^abc^	0.38 ^abcd^	3.67 ^bcd^
	Kapoor	0.56 ^bcdef^	0.23 ^bc^	0.79 ^cde^	0.32 ^bcde^	3.48 ^cde^
RB	Violeto	0.66 ^abc^	0.23 ^abc^	0.89 ^abcd^	0.43 ^ab^	4.36 ^a^
	Red Rubin	0.45 ^def^	0.17 ^c^	0.62 ^ef^	0.21 ^e^	3.94 ^b^
	Ablagh	0.58 ^bcde^	0.21 ^bc^	0.79 ^cde^	0.36 ^abcd^	4.54 ^a^
	Kapoor	0.57 ^bcdef^	0.20 ^bc^	0.77 ^cdef^	0.33 ^abcde^	4.28 ^a^
W	Violeto	0.70 ^abc^	0.22 ^bc^	0.91 ^abcd^	0.41 ^abc^	2.50 ^hi^
	Red Rubin	0.48 ^def^	0.18 ^bc^	0.66 ^ef^	0.26 ^de^	1.67 ^j^
	Ablagh	0.40 ^f^	0.16 ^c^	0.56 ^f^	0.20 ^e^	3.08 ^fg^
	Kapoor	0.53 ^cdef^	0.18 ^bc^	0.71 ^def^	0.30 ^cde^	2.42 ^i^
R	Violeto	0.59 ^bcd^	0.20 ^bc^	0.80 ^bcde^	0.45 ^a^	2.79 ^gh^
	Red Rubin	0.41 ^ef^	0.17 ^c^	0.57 ^f^	0.42 ^abc^	2.38 ^i^
	Ablagh	0.58 ^bcde^	0.19 ^bc^	0.77 ^cdef^	0.28 ^de^	3.33 ^def^
	Kapoor	0.54 ^cdef^	0.19 ^bc^	0.73 ^cdef^	0.38 ^abcd^	2.83 ^gh^
B	Violeto	0.82 ^a^	0.18 ^bc^	1.00 ^ab^	0.35 ^abcd^	3.15 ^efg^
	Red Rubin	0.79 ^a^	0.27 ^ab^	1.06 ^a^	0.21 ^e^	2.94 ^g^
	Ablagh	0.62 ^bcd^	0.21 ^bc^	0.82 ^bcde^	0.26 ^de^	3.81 ^bc^
	Kapoor	0.73 ^ab^	0.21 ^bc^	0.94 ^abc^	0.27 ^de^	3.30 ^ef^
Significance		**	*	**	**	**

Each number represent the average of four replicates (n = 80). ** Significant at *p* ≤ 0.01 and * Significant at *p* ≤ 0.05. Different letters within each column indicate significant differences according to Duncans multiple range test (*p* = 0.05). B: R (12/12 h) = Blue light 12 h + Red light 12 h, RB = Red + Blue continuous light, W = White continuous light, R = Red continuous light, B = Blue continuous light, Chl = Chlorophyll.

**Table 2:** Heat map correlation coefficient between studied characters.

	**B**	**Chl *a***	**Chl *b***	**Total Chl**	**CARs**	**TAC**	**TPC**	**TFC**	**Vit C**	**ACNs**	**APCI**	**VP**	**Nit**	**Pro**	**Carbo**	**Star**	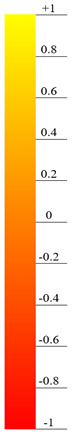
B		0.098	0.182	0.139	−0.018	0.298 *	−0.003	0.159	−0.132	0.091	0.156	0.852 **	0.423 **	−0.040	0.113	−0.112	
Chl *a*			0.358 **	0.951 **	0.218	−0.067	0.237	0.041	0.190	0.062	0.265 *	0.215	0.378 **	0.144	0.001	−0.531 **	
Chl *b*				0.628 **	0.203	−0.024	−0.009	0.150	0.119	0.135	0.217	0.262 *	0.087	0.074	0.209	−0.118	
Total Chl					0.254	−0.068	0.197	0.084	0.194	0.100	0.295 *	0.264 *	0.346 **	0.145	0.069	−0.479 **	
Cars						−0.239	0.157	0.277 *	0.112	−0.051	0.367 **	0.152	−0.290 *	0.241	0.193	0.287 *	
TAC							−0.125	−0.079	−0.084	0.160	0.114	0.312 *	0.284 *	−0.272 *	−0.058	−0.179	
TPC								0.688 **	0.642 **	0.072	0.855 **	0.437 **	0.201	0.830 **	0.225	−0.203	
TFC									0.469 **	0.013	0.825 **	0.535 **	−0.144	0.676 **	0.437 **	0.263 *	
Vit C										−0.133	0.639 **	0.271 *	−0.008	0.722 **	0.175	−0.192	
ACNs											0.102	0.121	0.260 *	0.045	0.145	−0.065	
APCI												0.638 **	0.178	0.776 **	0.416 **	−0.068	
VP													0.444 **	0.387 **	0.331 **	−0.140	
Nit														0.055	0.081	−0.603 **	
Pro															0.392**	−0.060	
Carbo																0.270 *	
Star																	

** Correlation is significant at the 0.01 level, * correlation is significant at the 0.05. n = 80, B = biomass; Chl *a* = Chlorophyll a; Chl *b* = Chlorophyll b; Total Chl = Total chlorophyll; CARs = Carotenoids; TAC = Total antioxidant capacity (%), TPC = Total phenolic compounds, TFC = Total flavonoid contents; Vit C = Vitamin C; ACNs = Anthocyanins; APCI: Antioxidant Potential Composite Index; VP: Value production; Nit: Nitrate; Pro: Proline; Carbo: Carbohydrates; Star: Starch.

**Table 3:** Mean comparison of antioxidants and biochemical compounds of basil microgreen cultivars under different LED light spectra.

Treatments		TAC (%DPPH Inhibition)	TPC (mg GA 100 g^−1^ FW)	TFC (mg CAE g^−1^ FW)	Vitamin C (mg 100 g^−1^ FW)	ACNs (mg 100 g^−1^ FW)	Nitrate (mg kg^−1^ FW)	Proline (mg g^−1^ FW)	Carbohydrate (g 100 g^−1^ FW)	Starch (mg g^−1^ FW)
Light Spectra	Cultivars									
B: R (12/12 h)	Violeto	78.35 ^a^	1170.33 ^de^	4.56 ^c^	174.48 ^ef^	23.70 ^ab^	1031.00 ^bc^	2.19 ^fgh^	5.35 ^ab^	8.62 ^bcde^
	Red Rubin	60.02 ^bcd^	900.69 ^ef^	2.14 ^gh^	165.88 ^f^	26.33 ^a^	726.82 ^fgh^	2.47 ^fgh^	5.52 ^a^	9.31 ^abc^
	Ablagh	52.43 ^de^	930.33 ^ef^	4.03 ^cd^	184.56 ^ef^	24.54 ^ab^	627.00 ^hji^	3.33 ^def^	4.13 ^cde^	7.71 ^efg^
	Kapoor	63.60 ^bc^	1000.45 ^def^	3.58 ^cde^	174.97 ^ef^	8.85 ^hi^	794.94 ^efg^	2.67 ^fgh^	5.00 ^abc^	8.55 ^bcdef^
RB	Violeto	48.24 ^ef^	1191.50 ^de^	5.85 ^b^	271.00 ^bcd^	18.98 ^e^	846.50 ^ef^	4.60 ^bc^	5.54 ^a^	9.14 ^abcd^
	Red Rubin	64.42 ^bc^	999.51 ^def^	1.82 ^gh^	153.29 ^f^	24.44 ^ab^	979.26 ^cd^	1.63 ^h^	1.79 ^k^	7.57 ^fgh^
	Ablagh	59.25 ^bcd^	1084.21 ^de^	2.72 ^efg^	203.65 ^def^	20.07 ^cde^	804.42 ^ef^	2.39 ^fgh^	2.50 ^hijk^	8.54 ^bcdef^
	Kapoor	57.31 ^cd^	1091.74 ^de^	3.46 ^def^	209.31 ^cdef^	10.16 ^h^	876.72 ^de^	2.87 ^efgh^	3.28 ^efghi^	8.41 ^cdef^
W	Violeto	53.07 ^de^	1017.86 ^def^	1.07 ^h^	279.81 ^bc^	15.41 ^fg^	546.31 ^ijk^	3.09 ^defg^	2.99 ^ghij^	6.83 ^ghi^
	Red Rubin	52.69 ^de^	1006.57 ^def^	2.50 ^fg^	263.44 ^bcd^	19.58 ^de^	672.00 ^ghi^	2.78 ^efgh^	3.51 ^defgh^	9.52 ^ab^
	Ablagh	67.13 ^b^	736.92 ^f^	1.71 ^gh^	179.31 ^ef^	14.71 ^g^	424.35 ^ki^	1.66 ^h^	1.97 ^jk^	8.17 ^def^
	Kapoor	57.63 ^cd^	920.45 ^ef^	1.76 ^gh^	240.85 ^bcde^	6.33 ^ij^	547.55 ^jik^	2.51 ^fgh^	2.82 ^ghij^	8.18 ^def^
R	Violeto	66.79 ^b^	1696.91 ^b^	1.24 ^h^	277.29 ^bc^	17.68 ^ef^	348.80 ^i^	3.96 ^cde^	3.05 ^fghi^	9.36 ^abc^
	Red Rubin	34.18 ^g^	1504.91 ^bc^	4.56 ^c^	239.27 ^bcde^	22.77 ^bc^	533.83 ^jk^	5.80 ^b^	4.10 ^cdef^	9.81 ^a^
	Ablagh	42.69 ^f^	1250.79 ^cd^	6.14 ^b^	285.31 ^b^	16.11 ^fg^	318.42 ^i^	4.28 ^de^	4.23 ^cde^	8.79 ^bcd^
	Kapoor	47.89 ^ef^	1484.20 ^bc^	3.98 ^cd^	267.30 ^bcd^	5.42 ^j^	400.35 ^i^	4.68 ^bc^	3.80 ^defg^	9.32 ^abc^
B	Violeto	60.99 ^bcd^	1079.98 ^de^	7.67 ^a^	169.45 ^ef^	22.39 ^bc^	1210.83 ^a^	1.95 ^gh^	2.39 ^ijk^	5.93 ^i^
	Red Rubin	40.79 ^fg^	1747.72 ^b^	4.38 ^cd^	272.25 ^bcd^	26.14 ^a^	1002.00 ^c^	4.81 ^bc^	3.74 ^defg^	6.71 ^hi^
	Ablagh	83.57 ^a^	2027.25 ^a^	5.74 ^b^	405.76 ^a^	23.78 ^ab^	1186.00 ^a^	7.47 ^a^	4.48 ^bcd^	6.06 ^i^
	Kapoor	43.78 ^f^	1618.32 ^b^	5.93 ^b^	282.49 ^bc^	7.83 ^hij^	1132.94 ^ab^	4.75 ^bc^	3.54 ^defgh^	6.24 ^i^
Significance		*	**	**	**	**	**	**	**	**

Each number represent the average of four replicates (n = 80), ** Significant at *p* ≤ 0.01 and * Significant at *p* ≤ 0.05. Different letters within each column indicate significant differences according to Duncan’s multiple range test (*p* = 0.05). B: R (12/12 h) = Blue light 12 h + Red light 12 h, RB = Red + Blue continuous light, W = White continuous light, R = Red continuous light, B = Blue continuous light, TAC = Total Antioxidants Capacity, TPC = Total phenolic compounds, TFC = Total flavonoid contents, ACNs = Anthocyanin, GA = Gallic acid, CAE = Catechin.

## Data Availability

Data will be made available on request.

## Supplementary Materials

The following supporting information can be downloaded at: https://www.mdpi.com/article/10.3390/plants13101394/s1.
