# Supplementary material for: Management of Secondary Metabolite Synthesis and Biomass in Basil (Ocimum basilicum L.) Microgreens Using Different Continuous-Spectrum LED Lights
Source: Plants (Basel). 2024 May 17;13(10):1394. doi: 10.3390/plants13101394 (PMC11125838; doi:10.3390/plants13101394)
Supplement: Supplementary file 1 [file plants-13-01394-s001.zip › plants-2979565-supplementary.pdf]

Table S1. Concentrations of Hoagland's nutrient solution (1938).

Consumption Nutrients	Concentration (mg L^{-1})	Consumption Nutrients	Concentration (mg L^{-1})
N	210	Fe	3
K	235	B	0.5
Ca	200	Mn	0.5
P	31	Zn	0.05
S	64	Cu	0.02
Mg	48	Mo	0.01
